# How perceived coercion polarizes unvaccinated people: The mediating role of conspiracy beliefs

**DOI:** 10.1177/13591053241238126

**Published:** 2024-03-17

**Authors:** Haiyan Wang, Jan-Willem van Prooijen, Paul AM van Lange

**Affiliations:** 1 Vrije Universiteit Amsterdam, The Netherlands; 2 The Netherlands Institute for the Study of Crime and Law Enforcement (NSCR), The Netherlands; 3 Maastricht University, The Netherlands

**Keywords:** conspiracy beliefs, polarization, vaccination coercion, vaccination status

## Abstract

During the COVID-19 pandemic, different policies were implemented to increase vaccination uptake. Meanwhile, conspiracy theories spread widely, and vaccinated versus unvaccinated people increasingly polarized against each other. This study examined the associations between perceived vaccination coercion, conspiracy beliefs and polarization. We tested the relationship of vaccination status with perceived vaccination coercion, conspiracy beliefs, and polarization, with a total sample size of *N* = 1202 (*n* = 400 in China, *n* = 401 in the US, and *n* = 401 in the UK), among them *n* = 603 were vaccinated and *n* = 599 were unvaccinated. As pre-registered, unvaccinated people perceived more vaccination coercion and endorsed more conspiracy theories. Conspiracy mentality was positively related to perceived coercion. Contrary to our hypotheses, vaccinated people were more polarized toward unvaccinated people than vice versa. Finally, conspiracy beliefs mediated the link between perceived coercion and polarization among unvaccinated people.

The COVID-19 pandemic has impacted people’s lives immensely. Vaccination was seen as the most efficient way to stop the spread of the coronavirus; however, not everyone was willing to get a COVID-19 vaccine (Hammer et al., 2021; Middleman et al., 2021; Ruiz and Bell, 2021). Many countries have installed policies at various points of the pandemic that were designed to curb the spread of the virus while encouraging citizens to get vaccinated, for example through a COVID-19 pass that one needed for access to public places. Citizens have perceived many of these policies as a form of coercion to get vaccinated, and conspiracy theories about the vaccines have spread rapidly (an “infodemic”; Zarocostas, 2020). Moreover, the vaccination campaigns have increased polarization between vaccinated and unvaccinated people (Bardosh et al., 2022; Bor et al., 2023; Henkel et al., 2022; MacDonald et al., 2022). For instance, vaccinated people have blamed unvaccinated people for being responsible for prolonging the pandemic, leading them to support more restrictive policies toward unvaccinated people (Schuessler et al., 2022); at the same time, many of these restrictive policies may be perceived as unfair and exclusionary by unvaccinated people. The present research examines what role conspiracy theories play in these processes, and how polarization is related to people’s vaccination status.

Conspiracy mentality is conceptualized as a general predisposition to interpret many events in the world as being caused by conspiracies, while specific conspiracy beliefs pertain to specific societal events (e.g. the pandemic) as being caused by a conspiracy (Imhoff et al., 2022). Those conspiracy theories are usually unsubstantiated. Although these constructs are highly correlated, and are even used interchangeably in many studies, their more specific effects may differ (e.g. Van Prooijen et al., 2023b). One noteworthy difference is that conspiracy mentality is more relatively stable over time while specific conspiracy beliefs are more malleable by situational factors (e.g. Stojanov et al., 2023; Stojanov and Halberstadt, 2020; Wang and Van Prooijen, 2023).

Previous research has revealed that conspiracy beliefs predict reduced COVID-19 preventive behaviors, such as keeping social distance and wearing face masks (Bierwiaczonek et al., 2020; Earnshaw et al., 2020; Imhoff and Lamberty, 2020; Jovančević and Milićević, 2020; Romer and Jamieson, 2020) and reduced COVID-19 vaccination intentions (Allington et al., 2021; Bacon and Taylor, 2021; Freeman et al., 2022; Garry et al., 2022; Winter et al., 2022). Conspiracy beliefs may even discourage families or friends from getting vaccinated (Freeman et al., 2022; see also Garry et al., 2022). However, much is still unclear about how these conspiracy beliefs have inspired polarization across societal groups throughout the vaccination campaign. Many media sources have reported tensions between vaccinated and unvaccinated people (Bielski, 2021; Ellyatt, 2021; Meissner, 2021; Nast, 2021). What role do conspiracy beliefs play in such polarization of people with a different vaccination status? To address these questions, we have examined the associations between perceived vaccination coercion, conspiracy beliefs about the COVID-19 vaccines, and polarization in three countries: the US, the UK, and China. Moreover, we have collected data to balance the number of vaccinated and unvaccinated citizens in our samples, to investigate the relationship of vaccination status with these variables.

## Conspiracy beliefs, vaccination hesitancy and vaccination status

As some vaccination policies have exerted pressure, particularly on people who were hesitant to get vaccinated, many of them might have felt some level of coercion to get vaccinated. This pressure may have stimulated people to justify their decision not to get vaccinated, making them sensitive to motivated reasoning processes (Kunda, 1990). Notably, the discrepancy between vaccination hesitancy and social norms of getting vaccinated during the pandemic may have prompted feelings of cognitive dissonance, stimulating motivated reasoning (Aronson, 1969; Festinger, 1957; Van Prooijen et al., 2023a).

Conspiracy beliefs can be part of such a motivated reasoning process, to serve ideological and psychological needs (Miller et al., 2016) and political interests (Enders and Smallpage, 2019). Motivated by the goal of not being vaccinated, people may become increasingly susceptible to misinformation (e.g. “vaccines are unsafe”) and conspiracy theories (Van der Linden, 2022). For instance, Howard and Davis (2023) found that conspiracy beliefs negatively predicted inoculation with the COVID-19 vaccines. As such, conspiracy beliefs help to legitimize people’s behavior that is perceived as counter-normative in the broader society (Van Prooijen, 2022). In a longitudinal study, Van Prooijen and Böhm (2023) found that vaccination hesitancy shapes increased conspiracy beliefs over time. Moreover, perceived vaccination coercion is likely to increase feelings of powerlessness and uncontrollability among vaccination-hesitant people. Such feelings of uncontrollability and lack of power are associated with increased conspiracy beliefs (Kofta et al., 2020; Kossowska and Bukowski, 2015; Van Prooijen and Acker, 2015; Van Prooijen and Van Lange, 2014). Based on these arguments, we hypothesize that unvaccinated people experience more vaccination coercion (Hypothesis 1a) and endorse stronger conspiracy beliefs about the COVID-19 vaccines (Hypothesis 1b).

While specific (e.g. vaccination) conspiracy beliefs are relevant in specific situations (e.g. a pandemic), conspiracy mentality indicates people’s general susceptibility to these conspiracy theories. The current study examined conspiracy mentality and COVID-19 vaccine conspiracy beliefs among groups of vaccinated and unvaccinated people. This actual vaccination status meaningfully extends research on vaccination intention given that intentions and actual behaviors do not always converge (e.g. Ajzen, 1991). For example, many people may be vaccination hesitant yet get vaccinated; likewise, some people may not be vaccination hesitant yet do not get vaccinated due to medical reasons. In light of the many studies that have found a relationship between vaccination hesitancy and conspiracy beliefs (Allington et al., 2021; Bacon and Taylor, 2021; Freeman et al., 2022; Garry et al., 2022; Winter et al., 2022), and taking into account that vaccination pass policies would limit the opportunities of only unvaccinated people, it stands to reason that unvaccinated people have a higher level of conspiracy mentality and experience more vaccination coercion. Hence, we hypothesize that conspiracy mentality is associated with perceived vaccination coercion (Hypothesis 2).

## Vaccination status and polarization

Vaccination status has divided people into two groups during the COVID-19 pandemic, and values about vaccination became polarized in both groups (Bardosh et al., 2022; Bor et al., 2023; Jost et al., 2022; MacDonald et al., 2022). Polarization is reflected in a strong conviction in the correctness of one’s beliefs, perceived moral superiority of one’s own values and beliefs, and conflict and hostility toward those who hold different values and beliefs (Van Prooijen, 2021). In the context of the COVID-19 vaccination campaign, vaccination statuses were particularly salient as a result of policies to reduce the spread of the corona virus. Given that people needed a vaccination pass to get access to public places with colleagues or friends, often it was clear who was vaccinated or not – this unlike other vaccines, where it usually is less clear to people who are and are not vaccinated.

Both vaccinated and unvaccinated people have shown ingroup favoritism, and were polarized in their attitudes and behaviors (Henkel et al., 2022). Before the pandemic, researchers noticed that both people who are pro-vaccination and anti-vaccination were polarized in their social media behaviors (e.g. likes, comments; see Schmidt et al., 2018). In addition, people are likely to receive information mainly from their “echo-chambers” (Van Bavel et al., 2020), which may increase polarization. Although both groups are likely to be polarized, evidence showed that vaccinated and unvaccinated people may not have polarized to an equal extent. For example, during the COVID-19 pandemic, Twitter conversations about vaccination were highly polarized especially among unvaccinated people (Hagen et al., 2021). Therefore, we predicted that unvaccinated people would be more polarized toward vaccinated people than vice versa (Hypothesis 3).^1^

While some vaccination policies may have increased uptake rate, some of these policies also may have stimulated conspiracy beliefs, especially considering that the COVID-19 vaccines are relatively new and have been developed in a short timeframe. To the extent that people experience vaccine policies as coercive, increased conspiracy beliefs about the COVID-19 vaccines might be endorsed to justify people’s vaccination hesitancy (Van Prooijen et al., 2023a; Van Prooijen and Böhm, 2023). Besides, people tend to expose themselves to information and people that validate their existing beliefs, contributing to group polarization (i.e. echo chambers; Choi et al., 2020; Cinelli et al., 2021; Del Vicario et al., 2016). Particularly unvaccinated people are likely to experience coercion in the vaccination campaign, however, and believe conspiracy theories that may contribute to polarization. We therefore predicted that perceived vaccination coercion promotes intergroup polarization, and that COVID-19 vaccination conspiracy beliefs mediate this effect. Moreover, we expect that vaccination status would moderate the links of perceived coercion with conspiracy belief and polarization, such that these links are significant and positive particularly among unvaccinated people (Hypothesis 4).

## The current study

Considering that policies differ across countries, we have collected data from three countries – the US, the UK, and China. In addition to participants’ perceived coercion to get vaccinated, we also measured people’s perceived coerciveness of policies, operationalized as how coercive people rate a standardized series of policies independent of whether they have been implemented in a particular country (as people may differ in how coercive they rate the same policy). In an exploratory fashion, we compared cultural differences for perceived coerciveness of policies as well.

## Method

### Participants

Within-country, we planned to compare the difference between vaccinated and unvaccinated people. A sample of 400 participants per country would generate a small to medium effect size (*d* = 0.28, power = 0.80, α = 0.05) for the within-country analyses. US and UK participants were recruited from Prolific (a platform that provides good data quality; see Peer et al., 2022), Chinese participants were recruited from Credamo (also a platform with good data quality; see Sheng et al., 2023). The total sample size was 1202 participants, with around 200 vaccinated and 200 unvaccinated participants from China, the UK and the US, respectively (for more information, see Online Supplemental materials Table S1). This study has been ethically approved by the Scientific and Ethical Review Board of the Faculty of Behavior and Movement Sciences (VCWE-S-21-00228[80]) at Vrije University Amsterdam. All the hypotheses were pre-registered on OSF: https://osf.io/4bcd8, Data are available via https://osf.io/gqj2s/.

### Procedure

The US and the UK reached a population-wide vaccination rate of 67% and 74%, respectively. China used another way of recording the vaccination rate, which was about 1.61 doses per citizen at the survey time. The data collection process started on October 25th, 2021 and ended on October 29th, 2021. Most citizens from these three countries had relatively little difficulty in getting access to the vaccine, as COVID-19 vaccines were widely available to the public (Mathieu et al., 2020). With the prescreening function offered by Prolific, we were able to selectively target vaccinated and unvaccinated participants in the UK and US. However, this prescreening information might be lagged, as some participants may have taken the vaccine without updating their information on Prolific. In order to reduce the influence of lagged prescreening, and accurately distinguish between vaccinated and unvaccinated participants in our sample, we designed two separate surveys in each country. In addition, we attached an alternative URL link that participants could choose, leading them to the correct survey based on their real vaccination status. Chinese participants were recruited from Credamo, which does not have the prescreening function. Instead, we indicated our target participants in the survey invitation as well as a question to double-check their vaccination status. Moreover, we randomly inserted an attention check (e.g. “Please choose Totally Disagree for this statement”), and a failed attention check would lead to the exclusion of a participant from the data.

### Measures

#### Perceived vaccination coercion

Perceived vaccination coercion was measured by asking participants to what extent they agreed with the statement “There is a lot of pressure from the government on people to get vaccinated” (“1 = Totally Disagree” to “7 = Totally Agree”).

#### Perceived coerciveness of policies

We asked participants to what extent they believed three measures are forcing people to get vaccinated (“1 = Not Forcing at All” to “7 = Very Much Forcing”) irrespective of whether those measures actually have been implemented in their country. For example, “Without being vaccinated, some employees are fired” (full scales of all measures can be found in the Online Supplemental materials). Cronbach’s α were α_China_ = 0.87, α_UK_ = 0.84, and α_US_ = 0.81 respectively.

#### Conspiracy mentality

We adopted the five-item measurement designed by Bruder et al. (2013), but measured on a seven-point scale (“1 = Totally Disagree” to “7 = Totally Agree”) to be consistent with other measurements, for example “Many important things happen in the world, which the public is never informed about.” Reliability in three countries were α_China_ = 0.88, α_UK_ = 0.83, and α_US_ = 0.84 respectively.

#### Conspiracy beliefs

Conspiracy beliefs were measured by asking participants to what extent they agreed with five conspiracy theories about the COVID-19 vaccines, for example, “The COVID-19 vaccines are used to manipulate citizens.” Participants rated each item on a scale from “1 = Totally Disagree” to “7 = Totally Agree.” The reliability of the conspiracy beliefs measurements in three countries were α_China_ = 0.83, α_UK_ = 0.94, and α_US_ = 0.96 respectively.

#### Polarization

After a short reminder of their vaccination status (“You have decided (not) to get vaccinated”), we measured polarization by asking participants to rate to what extent they agreed (“1 = Totally Disagree” to “7 = Totally Agree”) with nine statements about their personal conviction (e.g. “I believe my decision is the only right one”), moralization (e.g. “It is a moral obligation to promote the COVID-19 vaccine” for vaccinated participants and “It is a moral obligation to reject the COVID-19 vaccine” for unvaccinated participants) and perceived conflict (e.g. “I feel angry with unvaccinated people” for vaccinated participants and “I feel angry with vaccinated people” for unvaccinated people). These three elements are in line with the main aspects of polarization (Van Prooijen, 2021). The reliability of the polarization measurement in the three countries were α_China_ = 0.94, α_UK_ = 0.91, and α_US_ = 0.93 respectively.

## Results

To test Hypothesis 1a, Hypothesis 1b, and Hypothesis 3, we conducted a range of two-tailed *t*-tests between vaccinated and unvaccinated groups within each country. Hypothesis 2 was tested with a correlational analysis. Finally, we tested Hypothesis 4 with structural equation modeling. Age, educational background (1 = primary school, 5 = PhD degree), gender (male = −1, female = 1, others = *NA*) and political orientation (−50 = left-wing, 50 = right-wing) were included as control variables in the moderated mediation model analysis to avoid alternative explanations with demographic information and political orientation (Bernerth and Aguinis, 2016). As it is difficult to reliably measure political orientation in China, this variable was not measured among Chinese participants. As polarization is a multifaceted concept, we treated it as a latent variable. The structural equation model was analyzed with the R package “Lavaan” (Rosseel, 2012). Comparison analyses within-country were conducted with Jamovi (Version 1.6).

### Differences between vaccinated and unvaccinated groups

The associations of vaccination status with conspiracy mentality and beliefs, perceived coercion and polarization are shown in Online Supplemental materials Table S2. We found that both conspiracy mentality and conspiracy beliefs were significantly higher among the unvaccinated rather than the vaccinated group in the US, the UK and China (Figure S1 A and Figure S1 C in the Online Supplemental materials). As pre-registered (Hypotheses 1a and 1b), across cultures the unvaccinated group perceived higher vaccination coercion than the vaccinated group (Figure S1 B in Online Supplemental Material). Within cultures, both unvaccinated and vaccinated groups perceived high levels of vaccination coercion in the US and the UK (i.e. participants scored higher than the average value of 4 on a 7-point scale), but not in China. Contrary to our pre-registered Hypothesis 3, the vaccinated group was more polarized toward unvaccinated people than the unvaccinated group was toward vaccinated people in all three countries (Figure S1 D in Online Supplemental material).

**Figure 1.**
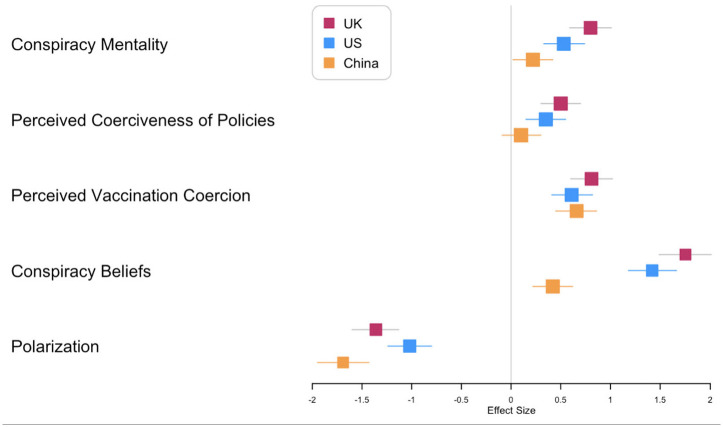
Effect sizes of the differences between the vaccinated and unvaccinated groups. Effect sizes are Cohen’s *d*. Effect size larger than 0 means that the unvaccinated group scored higher on a particular dependent variable, while an effect size smaller than 0 means that the vaccinated group scored higher.

We plotted effect size Cohen’s *d* and the 95% CI (calculated with the spreadsheet developed by Lakens, 2013; see https://osf.io/ixGcd/) in a forest plot (using the R package “forestplot”; Gordon and Lumley, 2021). The forest plot (Figure 1) shows a cross-culturally consistent effect, in that unvaccinated groups scored higher than vaccinated groups on conspiracy mentality, conspiracy beliefs and perceived vaccination coercion. With respect to conspiracy mentality and conspiracy beliefs, the effect of vaccination status appeared smaller in China than in the US and the UK. Interestingly, the effect of vaccination status on polarization was largest in China.

### Correlation analysis

A correlation analysis (Table S3 in Online Supplemental material) showed that both perceived vaccination coercion and perceived coerciveness of policies were positively correlated with conspiracy mentality and conspiracy beliefs, regardless of vaccination status. Thus, pre-registered Hypothesis 2 was supported. Polarization was positively correlated with perceived vaccination coercion among unvaccinated people, *r* = 0.25, *p*  <  0.001, while it was negatively correlated with perceived vaccination coercion among vaccinated people, *r* = −0.21, *p*  <  0.001. The correlations between polarization and conspiracy beliefs were also different between vaccinated and unvaccinated people: It was positive among unvaccinated people, *r* = 0.47, *p*  <  0.001, and negative among vaccinated people, *r* = −0.57, *p*  <  0.001.

### Moderated mediation analysis

**Figure 2.**
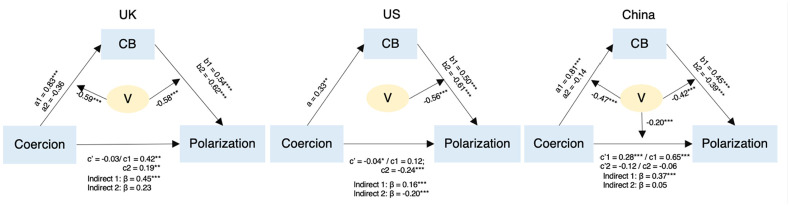
The effect of perceived vaccination coercion on polarization moderated by vaccination status. Coercion: perceived vaccination coercion; CB: conspiracy beliefs; V: vaccination status. All coefficients are β. Parameters for unvaccinated people are: a1, b1, c1, c’1, and indirect 1; parameters for vaccinated people are: a2, b2, c2, c’2, and indirect 2.

We first conducted a path analysis in each country to determine which paths are moderated by vaccination status (see Online Supplemental materials Table S4). We found moderated mediation effects in all three countries, however, there were differences in terms of the specific paths. Vaccination status moderated only the indirect effect in the US and the UK, but it moderated both the direct and indirect effects in China. These differences notwithstanding, our findings support the pre-registered hypothesis that the effect of perceived vaccination coercion on polarization was mediated by conspiracy beliefs, and this mediation was moderated by vaccination status. More precisely, there was a positive mediation effect among unvaccinated people in all three countries, while this mediation effect was negative among vaccinated people in the US but not in China and the UK (Figure 2).

In the US, perceived vaccination coercion predicted conspiracy beliefs (path *a* in the mediation model), but this effect was not moderated by vaccination status. However, this path was moderated by vaccination status in the UK and China: Perceived vaccination coercion positively predicted conspiracy beliefs among unvaccinated people, while this effect was not significant among vaccinated people (Table S5 in Online Supplemental material). These results indicate that in the US, perceiving stronger vaccination coercion predicts increased conspiracy beliefs regardless of participants’ vaccination status, whereas in the UK and China this effect only emerges among unvaccinated people.

In all three countries, conspiracy beliefs predicted polarization against the opposite vaccination status group (path *b* in the mediation model), and this effect was moderated by vaccination status. These results suggest that cross-culturally, conspiracy beliefs predict increased polarization among unvaccinated people; interestingly, *dis*belief in conspiracy theories predicts increased polarization among vaccinated people.

### Perceived coerciveness of policies

In a more exploratory fashion, we also conducted a 3 (Country: US; UK; China) × 2 (Vaccination Status: Vaccinated; Unvaccinated) ANOVA (Welch’s) analysis on people’s perceived coerciveness of a range of standardized policies. A main effect of country indicated that people differed in the extent to which they considered a range of prespecified policies coercive in the three countries, *F*(2, 1198) = 21.62, *p*  <  0.001, ω^2^ = 0.02. The main effect of vaccination status was significant as well, *F* (1, 1198) = 27.77, *p*  <  0.001, ω^2^ = 0.03. We then conducted post-hoc comparisons between countries (see Online Supplemental materials Table S6) and found that the perceived coerciveness of policies was significantly lower in China as compared to the UK and the US; however, there was no difference between the UK and the US. The effects of vaccination status on perceived coerciveness of policies across the three countries were different (Figure 1): Perceived coerciveness of policies was higher among unvaccinated than vaccinated people in the UK and the US, but not in China.

## Discussion

This study investigated the effects of vaccination status cross-culturally. Hypotheses 1a, 1b, and 2 were supported as preregistered. Hypothesis 3 was not supported, however, and results in fact revealed the opposite (vaccinated people were more polarized toward unvaccinated people than vice versa). We also tested the mediating role of conspiracy beliefs in the link between perceived vaccination coercion and polarization among vaccinated versus unvaccinated people. While there were some unexpected differences between countries, in all three countries the moderated mediation model analysis revealed that perceived vaccination coercion predicts increased belief in vaccination conspiracy theories; these conspiracy beliefs, in turn, are associated with increased polarization among unvaccinated people. These findings support Hypothesis 4.

The mediation model indicates that conspiracy beliefs play a crucial role in the polarization of unvaccinated people as a function of perceived coercion to get vaccinated. For vaccinated people, perceived vaccination coercion was unrelated to polarization. Moreover, conspiracy beliefs predicted *reduced* polarization among vaccinated people. This latter finding may suggest that the more strongly vaccinated people reject conspiracy theories, the more strongly they also reject unvaccinated people. However, this mediation model among vaccinated people is less robust than for unvaccinated people, as it fits only in the US, but not in the UK and China.

A notable finding of this study is that vaccinated people were more polarized than unvaccinated people, contradicting Hypothesis 3. Although unexpected, this finding is consistent with an earlier finding that people with low levels of COVID-19 conspiracy beliefs are more likely to end contact with conspiracy believers rather than vice versa (Van Prooijen, 2021). Similarly, Bor et al. (2023) found that vaccinated people have stronger discriminatory attitudes toward unvaccinated people than vice versa. Also, cognitive dissonance could be a factor as people may justify their decision of not being vaccinated to be reasonable. This may have decreased levels of polarization among the unvaccinated.

In an exploratory fashion, we distinguished between perceived vaccination coercion in real life and perceived coerciveness of policies, in consideration of cultural differences in health-related policy perceptions (Sabat et al., 2020; Von Lengerke et al., 2004). We specifically sought to consider that (a) within a culture people may differ in how coercive they experience the same set of measures but also that (b) different countries may have different policies. As it turned out, the Chinese sample perceived less vaccination coercion than the UK and the US samples, but that does not mean the measures were less strict in China: Also perceived coerciveness of policies (i.e. participants’ ratings of how coercive a standardized set of policies were independent of whether they were implemented or not) was much lower in China than in the UK and the US. Although speculative, this difference might be explained by the “tightness versus looseness” cultural dimension: Being a typical “tight” culture, China has stricter social norms and punishments (Gelfand et al., 2011), and this may lead people to be relatively tolerant of strict governmental policies. Another explanation might be that the Chinese had a relatively high acceptance of COVID-19 vaccines (Lazarus et al., 2021), implying lower levels of perceived coercion. These findings are noteworthy also for policymakers, as they might imply that some measures to stimulate vaccination may work better in some countries than others.

It is important to note that unvaccinated people are not necessarily “anti-vaxxers.” For example, some people might have decided not to be vaccinated for medical reasons. People may have different motives for accepting or refusing vaccination, and therefore we have avoided overinterpretations about people’s underlying motives based on their vaccination status.

### Implications

The risks of a COVID-19 infection differ across people; for example, elderly people are more likely to have life-threatening symptoms when they are infected. The COVID-19 pandemic therefore has created a complex situation in which governments had to deal with a multi-level social dilemma between collective versus individual interests (Van Lange and Rand, 2022). Many countries have resorted to policies which could be construed as coercive, but that are likely to have increased vaccination uptake. Our findings suggest that these measures also may have had a downside, as they predict conspiracy beliefs and polarization especially among unvaccinated people. This is likely to have downstream implications for public health. Jensen et al. (2021) found that believing in COVID-19 vaccine conspiracies were related to reduced trust in science and official information. In the vaccination campaign, more objective arguments other than moral persuasion strategies therefore should be applied to reduce societal polarization (Abeywickrama et al., 2020). To increase the uptake of vaccination rate, people need easy access not only to the vaccine, but also to trustworthy information that prebunks and debunks conspiracy theories (Van der Linden, 2022). For example, people should be encouraged to discuss COVID-19 vaccines with their healthcare provider when they are hesitant about vaccination, given that people generally perceive healthcare providers as more trustworthy than politicians (Sparks et al., 2022). In addition, scientific communication about vaccination safety is more effective in overcoming hesitancy than imposing coercive policies (Mello et al., 2022).

### Strengths and limitations

This study has various strengths and limitations. Among the strengths are that we collected data in three countries, including a non-WEIRD society (China). Indeed, there were cultural differences for some of the results in this study, especially China as compared with the US and UK. This suggests that in future research, more cross-cultural studies about the link between conspiracy beliefs and health behavior are needed. Furthermore, although we present only one study, it had a strong methodology: We preregistered this study, conducted it in three different countries, and it was well-powered with a big sample size. These methodological features suggest that the results are reliable. Finally, by contrasting people with a different vaccination status, this study offers clear relevance for public health and the consequences of public health policies.

There are also noteworthy limitations of this study. One issue is that the samples in each country were not representative for their population. For example, elderly and sick people who benefited tremendously from the vaccine were underrepresented. Also, the reliability of the conspiracy belief measures is different across the three countries, which suggests that conspiracy beliefs may be to some degree culture specific; moreover, we should highlight that the conspiracy belief measure consisted of self-designed items. It is important to acknowledge the possibility that cultural differences, or differences in policies between nations, can influence the nature of the conspiracy theories, and therefore the specific meaning of the items. Moreover, this is a cross-sectional study, and hence does not show the causality of the relations investigated here. This makes it also difficult to control for common method variance, which may inflate the effect sizes.

In addition, polarization is a complex construct, and it is difficult to capture the nature of polarization in one single measure. As polarization is often domain-specific, precise measurements are always a challenge in this research area. Moreover, we used a single item to measure perceived vaccination coercion, and future research would benefit from more items that complement each other (e.g. by tapping various forms of coercion that people may perceive). Finally, our study focused on understanding polarization among unvaccinated people; however, our results suggested that vaccinated people are more polarized toward unvaccinated people than vice versa. More research is needed to explain and replicate this finding, also in the aftermath of the COVID-19 pandemic.

## Concluding remarks

Perceived vaccination coercion is associated with increased vaccination-related conspiracy beliefs and polarization among unvaccinated people. The current findings suggest that policy makers faced a trade-off between public health and social cohesion during the COVID-19 pandemic. Both the benefits and drawbacks of potentially coercive vaccination measures should be considered in decision-making around this precarious issue. Not only the coronavirus, but also health-related conspiracy theories are harmful to public health and might damage social cohesion. Therefore, vaccination policies should be implemented carefully, to reduce the risk of replacing an epidemic for an infodemic.

## Supplemental Material

sj-docx-1-hpq-10.1177_13591053241238126 – Supplemental material for How perceived coercion polarizes unvaccinated people: The mediating role of conspiracy beliefs Supplemental material, sj-docx-1-hpq-10.1177_13591053241238126 for How perceived coercion polarizes unvaccinated people: The mediating role of conspiracy beliefs by Haiyan Wang, Jan-Willem van Prooijen and Paul AM van Lange in Journal of Health Psychology

## Notes

1. Note that Hypotheses 3 and 4 in the paper are listed as Hypotheses 1c and 3 in the pre-registration.
