# Supplementary material for: How perceived coercion polarizes unvaccinated people: The mediating role of conspiracy beliefs
Source: J Health Psychol. 2024 Mar 17;30(9):2354–67. doi: 10.1177/13591053241238126 (PMC12322336; doi:10.1177/13591053241238126)
Supplement: sj-docx-1-hpq-10.1177_13591053241238126 – Supplemental material for How perceived coercion polarizes unvaccinated people: The mediating role of conspiracy beliefs [file sj-docx-1-hpq-10.1177_13591053241238126.docx]

Online Supplemental Materials for

**How Perceived Coercion Polarizes Unvaccinated People: The Mediating Role of Conspiracy Beliefs**

**Measures**

Perceived Vaccination Coercion

We originally had measured perceived vaccination coercion with three items: “1 - There is a lot of pressure from the government on people to get vaccinated”, “2 - Life is getting increasingly difficult if people choose not to get vaccinated” and “3 - I feel that getting vaccinated or not is not really a choice anymore”. However, these three items generated low reliability, especially among samples from UK and US (α_China_ = .74, α_UK_ = .64, and α_US_ = .42). According to the concept we are measuring, Item-1 provides the best construct validity. We hence took this item as the measurement of perceived vaccination coercion.

***Perceived Coerciveness of Policies***

Countries differ in their vaccination policies. Here we will present to you measures that have been taken in some countries. Irrespective of whether it has or has not been implemented in your country, our question is to what extent you feel that these measures are forcing people to be vaccinated?

1. Without being vaccinated, some employees are fired;
2. Almost everywhere people go, a vaccination pass is required;
3. Companies require employees to be fully vaccinated in order to work in office.

***Conspiracy Mentality***

1. Many important things happen in the world, which the public is never informed about;
2. Politicians usually don't tell us the true motives for their decisions;
3. Government agencies closely monitor all citizens;
4. Events which superficially seem to lack a connection are often the result of secret activities;
5. There are secret organizations that greatly influence political decisions.

***Conspiracy Beliefs***

1. The COVID-19 vaccines are used to manipulate citizens;
2. The COVID-19 vaccination campaign serves to enable the government to blame the pandemic on unvaccinated people;
3. The COVID-19 vaccination campaign is not about public health but about a small group of powerful people making a lot of money;
4. Pharmaceutical companies lie about the possible dangers and side-effects of the COVID-19 vaccines;
5. The COVID-19 vaccine was developed with a hidden and immoral agency;

***Polarization***

You have decided (not) to get vaccinated. Now, please answer the following questions:

Conviction:

1. I believe this is the right decision;
2. I believe my decision is the only right one;
3. I believe everyone should make the same decision as I did about vaccination.

Moralization:

1. My decision to (not) get vaccinated is the only morally right one;
2. It is a moral obligation to promote / reject the COVID-19 vaccine;
3. I feel morally superior to unvaccinated / vaccinated people

Conflict:

1. I feel angry with unvaccinated / vaccinated people;
2. I prefer to keep distance from people who are unvaccinated / vaccinated;
3. I often feel offended by unvaccinated / vaccinated people.

**Table S1**

*Information about Participants in Each Country*

| Country | *N* | Age (*SD*) | *n* | | Gender ^a^ | |
| --- | --- | --- | --- | --- | --- | --- |
|  |  |  | Vaccinated | Unvaccinated | Male | Female |
| China | 400 | 29.85 (6.54) | 200 | 200 | 169 | 231 |
| UK | 401 | 31.44 (9.72) | 203 | 198 | 201 | 198 |
| US | 401 | 32 (10.07) | 200 | 201 | 197 | 199 |
| Total | 1202 | - | 603 | 599 | 567 | 628 |

*Note.* ^a^ There were three categories of gender in our survey: Male, Female, and Others. There were only seven (two from UK, five from US) participants who described themselves as “Others”, which were treated as missing data in the analyses.

**Table S2**

*Comparison between Vaccinated and Unvaccinated Groups Across Countries*

| Group |  | unvaccinated | | | vaccinated | | | *t* | *df* | *p* | Cohen’s *d* |
| --- | --- | --- | --- | --- | --- | --- | --- | --- | --- | --- | --- |
|  |  | *n* | *M* | *SD* | *n* | *M* | *SD* |  |  |  |  |
| Conspiracy Mentality | US | 201 | 5.6 | 1.1 | 200 | 4.7 | 1.2 | 7.98 | 399 | <.001 | 0.80 |
|  | UK | 198 | 5.5 | 1.0 | 203 | 4.9 | 1.1 | 5.34 | 399 | <.001 | 0.53 |
|  | China | 200 | 4.1 | 1.4 | 200 | 3.8 | 1.5 | 2.21 | 398 | .028 | 0.22 |
| Perceived Vaccination Coercion | US | 201 | 6.4 | 1.2 | 200 | 5.2 | 1.6 | 8.10 | 399 | <.001 | 0.81 |
|  | UK | 198 | 6.3 | 1.3 | 203 | 5.4 | 1.5 | 6.13 | 399 | <.001 | 0.61 |
|  | China | 200 | 4.0 | 1.7 | 200 | 3.0 | 1.6 | 6.56 | 398 | <.001 | 0.66 |
| Conspiracy Beliefs | US | 201 | 4.8 | 1.6 | 200 | 2.1 | 1.4 | 17.54 | 399 | <.001 | 1.75 |
|  | UK | 198 | 4.7 | 1.6 | 203 | 2.5 | 1.5 | 14.25 | 399 | <.001 | 1.42 |
|  | China | 200 | 2.2 | 1.2 | 200 | 1.8 | 0.7 | 4.17 | 398 | <.001 | 0.42 |
| Polarization | US | 201 | 2.7 | 1.1 | 200 | 4.6 | 1.5 | -13.66 | 399 | <.001 | -1.36 |
|  | UK | 198 | 2.8 | 1.2 | 203 | 4.1 | 1.4 | -10.24 | 399 | <.001 | -1.02 |
|  | China | 200 | 2.6 | 1.4 | 200 | 4.7 | 1.0 | -16.92 | 398 | <.001 | -1.69 |

H_0_ μ _unvaccinated_ ≠ μ _vaccinated_

**Table S3**

|  | 1 | 2 | 3 | 4 | 5 |
| --- | --- | --- | --- | --- | --- |
| 1. Perceived Coerciveness of Policies | - | .28*** | .26*** | .23*** | -.11** |
| 2. Perceived Vaccination Coercion | .39*** | - | .34*** | .30*** | -.21*** |
| 3. Conspiracy Mentality | .33*** | .56*** | - | .44*** | -.26*** |
| 4. Conspiracy Beliefs | .32*** | .61*** | .71*** | - | -.57*** |
| 5. Polarization | .13** | .25*** | .37*** | .47*** | - |

*The Correlations between Study Variables*

*Note.* The results for the unvaccinated sample (*n* = 599) are shown below the diagonal. The results for the vaccinated sample (*n* = 603) are shown above the diagonal.

**p* < .05. ***p* < .01. ****p* < .001.

**Table S4**

*Regressions of Moderated Mediation Model in US, UK and China (Three paths)*

|  | Mediation Variable: Conspiracy Beliefs | | | | | | | | | | | | | | |  |
| --- | --- | --- | --- | --- | --- | --- | --- | --- | --- | --- | --- | --- | --- | --- | --- | --- |
|  | UK | | | | | US | | | | | China | | | | | |
|  | *B* | *SE* | β | *p* | 95% CI | *B* | *SE* | β | *p* | 95% CI | *B* | *SE* | β | *p* | 95% CI |  |
| Age | 0.01 | 0.01 | 0.04 | .297 | [-0.01, 0.02] | 0.02 | 0.01 | 0.08 | .024 | [0.00, 0.03] | 0.01 | 0.01 | 0.06 | .168 | [-0.00, 0.02] |  |
| Edu | -0.13 | 0.10 | -0.05 | .197 | [-0.33, 0.07] | -0.12 | 0.09 | -0.05 | .193 | [-0.30, 0.06] | 0.27 | 0.10 | 0.13 | .004 | [0.09, 0.48] |  |
| Gen | 0.18 | 0.07 | 0.10 | .01 | [0.04, 0.32] | 0.16 | 0.07 | 0.08 | .015 | [0.03, 0.29] | -0.09 | 0.05 | -0.09 | .042 | [-0.18, -0.00] |  |
| PO | 0.02 | 0.00 | 0.23 | < .001 | [0.01, 0.03] | 0.02 | 0.00 | 0.30 | < .001 | [0.02, 0.03] | - | - | - | - | - |  |
| Co (a) | **0.30** | **0.05** | **0.23** | **< .001** | **[[0.20, 0.39]** | **0.31** | **0.05** | **0.23** | **<.001** | **[0.21, 0.40]** | **0.20** | **0.03** | **0.34** | **< .001** | **[0.14, 0.25]** |  |
| **Co*Vac** | **-0.19** | **0.05** | **0.59** | **< .001** | **[-0.28, 0.09]** | 0.06 | 0.05 | -0.18 | .201 | [-0.16, 0.03] | **-0.12** | **0.03** | **-0.47** | **<.001** | **[-0.18, -0.07]** |  |
|  | Dependent Variable: Polarization | | | | | | | | | | | | | | |  |
| Age | 0.00 | 0.02 | 0.00 | .227 | [-0.00, 0.01] | -0.00 | 0.00 | -0.02 | .278 | [-0.01, 0.00] | 0.02 | 0.01 | 0.05 | .013 | [0.00, 0.04] |  |
| Edu | 0.13 | 0.05 | 0.04 | .017 | [0.02, 0.23] | -0.02 | 0.04 | -0.01 | .724 | [-0.10, 0.07] | -0.16 | 0.11 | -0.03 | .144 | [-0.38, 0.06] |  |
| Gen | -0.04 | 0.04 | -0.02 | .334 | [-0.11, 0.04] | 0.02 | 0.03 | 0.01 | .567 | [-0.04, 0.08] | -0.04 | 0.05 | -0.01 | .466 | [-0.14, 0.06] |  |
| PO | -0.00 | 0.00 | -0.00 | .949 | [-0.00, 0.00] | -0.00 | 0.00 | -0.05 | .004 | [-0.01, -0.00] | - | - | - | - | - |  |
| Co (c’) | -0.05 | 0.03 | -0.03 | .053 | [-0.11, 0.00] | **-0.06** | **0.02** | **0.04** | **.019** | **[-0.011 0.01]** | **0.13** | **0.03** | **0.08** | **< .001** | **[0.07, 0.20]** |  |
| CB (b) | -0.04 | 0.03 | -0.04 | .098 | 1.02, 1.78] | **-0.06** | **0.02** | **-0.06** | **.017** | **[-0.10, -0.01]** | **0.09** | **0.06** | **0.03** | **.156** | **[0.03, 0.21]** |  |
| Co*Vac | 0.02 | 0.03 | 0.07 | .376 | [-0.03, 0.08] | 0.01 | 0.02 | 0.04 | .599 | [-0.03, 0.06] | **-0.14** | **0.03** | **-0.20** | **<.001** | **[-0.21, -0.08]** |  |
| CB*Vac | **-0.32** | **0.04** | **-0.58** | **< .001** | **[-0.39, -0.25]** | **-0.30** | **0.03** | **-0.55** | **< .001** | **[-0.36, -0.23]** | **-0.53** | **0.06** | **-0.42** | **< .001** | **[-0.65, -0.41]** |  |

*Note.* PO: political orientation; Co: perceived real-life coercion; CB: conspiracy beliefs; Unv: unvaccinated; Vac: vaccinated.

**p* < .05. ***p* < .01. ****p* < .001.

**Table S5**

*Moderated Mediation Model in US, UK and China*

|  | Mediation Variable: Conspiracy Beliefs | | | | | | | | | | | | | | |  |
| --- | --- | --- | --- | --- | --- | --- | --- | --- | --- | --- | --- | --- | --- | --- | --- | --- |
|  | UK | | | | | US | | | | | China | | | | | |
|  | *B* | *SE* | β | *p* | 95% CI | *B* | *SE* | β | *p* | 95% CI | *B* | *SE* | β | *p* | 95% CI |  |
| Age | 0.01 | 0.01 | 0.04 | .297 | [-0.01, 0.02] | 0.01 | 0.01 | 0.06 | .101 | [-0.00, 0.03] | 0.01 | 0.01 | 0.06 | .168 | [-0.00, 0.02] |  |
| Edu | -0.13 | 0.10 | -0.05 | .197 | [-0.33, 0.07] | -0.39 | 0.10 | -0.15 | < .001 | [-0.60, -0.19] | 0.29 | 0.10 | 0.13 | .004 | [0.09, 0.48] |  |
| Gen | 0.18 | 0.07 | 0.10 | .010 | [0.04, 0.32] | 0.20 | 0.08 | 0.10 | .009 | [0.05, 0.35] | -0.09 | 0.05 | -0.09 | .042 | [-0.18, -0.00] |  |
| PO | 0.02 | 0.00 | 0.23 | < .001 | [0.01, 0.03] | 0.03 | 0.00 | 0.44 | < .001 | [0.03, 0.04] | - | - | - | - | - |  |
| Co (a) | **0.30** | **0.05** | **0.23** | **< .001** | **[0.20, 0.40]** | **0.44** | **0.05** | **0.33** | **<.001** | **[0.33, 0.54]** | **0.20** | **0.03** | **0.34** | **< .001** | **[0.14, 0.25]** |  |
| Co*Vac | **-0.19** | **0.05** | **-0.59** | **< .001** | **[-0.28, -0.09]** | - | - | - | - | - | **-0.12** | **0.03** | **-0.47** | **< .001** | **[-0.18, -0.07]** |  |
| Conditional |  |  |  |  |  |  |  |  |  |  |  |  |  |  |  |  |
| **Co-Unv** | **0.48** | **0.08** | **0.83** | **< .001** | **[0.34, 0.63]** | - | - | - | - | - | **0.32** | **0.04** | **0.81** | **< .001** | **[0.25, 0.40]** |  |
| Co-Vac | 0.11 | 0.07 | -0.36 | .088 | [-0.02, 0.24] | - | - | - | - | - | 0.07 | 0.04 | -0.14 | .067 | [-0.01, 0.15] |  |
|  | Dependent Variable: Polarization | | | | | | | | | | | | | | |  |
| Age | 0.00 | 0.00 | 0.02 | .233 | [-0.00, 0.01] | -0.00 | 0.00 | -0.02 | .280 | [-0.01, 0.00] | 0.02 | 0.01 | 0.05 | .013 | [0.00, 0.04] |  |
| Edu | 0.13 | 0.05 | 0.04 | .017 | [0.02, 0.23] | -0.02 | 0.04 | -0.01 | .721 | [-0.10, 0.07] | -0.16 | 0.11 | -0.03 | .144 | [-0.38, 0.06] |  |
| Gen | -0.04 | 0.04 | -0.02 | .292 | [-0.11, 0.03] | 0.02 | 0.03 | 0.01 | .566 | [-0.04, 0.08] | -0.04 | 0.05 | -0.01 | .466 | [-0.14, 0.06] |  |
| PO | 0.00 | 0.00 | 0.00 | .977 | [-0.00, 0.00] | -0.00 | 0.00 | -0.05 | .004 | [-0.01, -0.00] | - | - | - | - | - |  |
| Co (c’) | -0.05 | 0.03 | -0.03 | .074 | [-0.10, 0.01] | **0.05** | **0.02** | **-0.04** | **.021** | **[-0.10, 0.10]** | **0.13** | **0.03** | **0.08** | **< .001** | **[0.07, 0.20]** |  |
| CB (b) | -0.05 | 0.03 | -0.03 | .074 | [-0.10, 0.01] | **-0.06** | **0.02** | **-0.06** | **.016** | **[-0.10, -0.01]** | 0.09 | 0.06 | 0.03 | .156 | [-0.03, 0.21] |  |
| Co*Vac | - | - | - | - | - | - | - | - | - | - | **-0.14** | **0.03** | **-0.20** | **< .001** | **[-0.21, -0.08]** |  |
| CB*Vac | **-0.32** | **0.03** | **-0.58** | **< .001** | **[-0.38, -0.25]** | **-0.29** | **0.03** | **-0.56** | **< .001** | **[-0.35, -0.23]** | **-0.53** | **0.06** | **-0.42** | **< .001** | **[-0.65, -0.41]** |  |
| Conditional |  |  |  |  |  |  |  |  |  |  |  |  |  |  |  |  |
| Co-Unv | - | - | - | - | - | - | - | - | - | - | **0.28** | **0.05** | **0.28** | **< .001** | **[0.18, 0.37]** |  |
| Co-Vac | - | - | - | - | - | - | - | - | - | - | -0.01 | 0.05 | -0.12 | .806 | [-0.10, 0.08] |  |
| CB-Unv | **0.27** | **0.04** | **0.54** | **< .001** | **[0.19,0.35]** | **0.24** | **0.04** | **0.50** | **< .001** | **[0.17, 0.31]** | **0.61** | **0.07** | **0.45** | **< .001** | **[0.48, 0.75]** |  |
| CB-Vac | **-0.36** | **0.05** | **-0.62** | **< .001** | **[-0.45, -0.27]** | **-0.35** | **0.04** | **-0.61** | **< .001** | **[-0.43, -0.27]** | **-0.44** | **0.10** | **-0.39** | **< .001** | **[-0.64, -0.24]** |  |
| ind-Unv | **0.13** | **0.03** | **0.45** | **< .001** | **[0.08, 0.18]** | **0.10** | **0.02** | **0.16** | **< .001** | **[0.06, 0.14]** | **0.20** | **0.03** | **0.37** | **< .001** | **[0.13, 0.26]** |  |
| ind-Vac | -0.04 | 0.02 | 0.23 | .095 | [-0.09, 0.01] | **-0.15** | **0.03** | **-0.20** | **< .001** | **[-0.20, -0.10]** | -0.03 | 0.02 | 0.05 | .092 | [-0.07, 0.01] |  |
| tot-Unv | 0.08 | 0.03 | 0.41 | .017 | 0.02, 0.15] | 0.05 | 0.03 | 0.12 | .070 | [-0.00, 0.10] | **0.47** | **0.05** | **0.65** | **< .001** | **[0.38, 0.57]** |  |
| tot-Vac | **-0.09** | **0.04** | **0.19** | **.014** | **[-0.16, -0.02]** | **-0.21** | **0.03** | **-0.24** | **< .001** | **[-0.27, -0.14]** | -0.04 | 0.05 | -0.06 | .365 | [-0.14, 0.05] |  |

*Note.* PO: political orientation; Co: perceived real-life coercion; CB: conspiracy beliefs; Unv: unvaccinated; Vac: vaccinated.

**p* < .05. ***p* < .01. ****p* < .001.

**Table S6**

*The Post Hoc Comparisons between Countries for Perceived Coerciveness of Policies*

| Comparison | | *MD* | *SE* | *df* | *t* | *p_holm_* | Cohen’s *d* |
| --- | --- | --- | --- | --- | --- | --- | --- |
| China | UK | -0.69 | 0.11 | 1196 | -6.10 | <.001 | -0.43 |
|  | US | -0.59 | 0.11 | 1196 | -5.21 | <.001 | -0.37 |
| UK | US | 0.10 | 0.11 | 1196 | 0.89 | .373 | 0.06 |

**Figure S1**

*Differences between Vaccinated and Unvaccinated Groups*

| A:  ***  *** | ***  ***  B: |
| --- | --- |
| D:  C:  ***  ***  *** | ***  ***  *** |

*Note. *p* < .05. ***p* < .01. ****p* < .001.
